# How DASH enables external data linkage to support multi-regional research

**DOI:** 10.23889/ijpds.v9i5.2503

**Published:** 2024-09-18

**Authors:** Ali Anis, Carrie-Anne Whyte, Carmen La, Jean-François Ethier, Mark McGilchrist

**Affiliations:** 1 Institute for Health Information; 2 Université de Sherbrooke; 3 University of Dundee

A network of organizations works together to support multi-regional health research across Canada. The network comprises 13+ provincial/territorial and pan-Canadian data centres, which collectively hold 500+ data assets. Although the network actively pursues new administrative or clinical data assets, linking to external research data is also a growing need in the contemporary research landscape.

Data from the network’s centres can be linked to external data sources such as that from: researchers’ trials or studies; disease or population-based registries; and data sources from other organizations or custodians. Consultations held with data centers clarified their processes for linking to external data, by identifying and mapping local linkage features to a general linkage model.

Local processes for data linkage, including necessary agreements and approval steps are now modelled and documented, and available to researchers and the data centres as a resource. This information helps streamline data access and linkages to data assets across the network and externally. Operationally, the network is currently working on 11 data access requests involving linkage to external data, of which three are expected to deliver final data to researchers by spring 2024.

Collaboration with data centres, affiliated organizations, and researchers are foundational in the development of linkage models across the network. These models play a critical role in making linkages across data sources within Canada more efficient and standardized. Data linkages across data assets support the utility of data and health innovation.

